# Oropharyngeal Secondary Syphilis Mimicking Metastatic Lymphoma in an HIV-Positive Patient

**DOI:** 10.7759/cureus.38072

**Published:** 2023-04-24

**Authors:** Tyler J Ostrowski, Maria Faraz, Matthew Holdaway, Anne Chen, Neil Gildener-Leapman

**Affiliations:** 1 Department of Otolaryngology - Head and Neck Surgery, Albany Medical Center, Albany, USA; 2 Department of Pathology and Laboratory Medicine, Albany Medical Center, Albany, USA

**Keywords:** biopsy, infectious disease, syphilis, head and neck, lymphoma

## Abstract

Syphilis is a bacterial infection commonly transmitted by sexual contact. It has variable manifestations and can mimic other disease processes or infections. This report presents the case of a 48-year-old HIV-positive male who was referred to our head and neck clinic with complaints of tonsillar hypertrophy and ulceration accompanied by a one-month history of ipsilateral cervical lymphadenopathy and facial pain in the setting of recent unexplained weight loss and abnormal radiographic imaging of the neck. In-office tonsillar biopsy and fine-needle aspiration of a neck mass revealed a non-diagnostic atypical lymphoid proliferation. Surgical pathology following an open biopsy in the operating room showed *Treponema pallidum* infection, which was diagnostic for secondary syphilis.

## Introduction

Syphilis is a sexually transmitted bacterial infection caused by the spirochete* Treponema pallidum* [1]. In the medical and scientific community, syphilis is known as *the great imitator* for its ability to mimic various other infectious and non-infectious conditions due to its many stages and wide range of clinical manifestations [2]. The stages of syphilis, which include primary, secondary, latent, and tertiary, are defined by various characteristic findings. Of all the stages, secondary syphilis presents with the most variable and ambiguous findings such as a skin rash, hepatosplenomegaly, lymphadenopathy, and mucosal ulceration, as well as a host of constitutional symptoms [2,3]. Due to its enigmatic nature, it is imperative that physicians keep this on their differential when evaluating patients, especially those who are sexually active. Although the prevalence of primary and secondary syphilis was at its nadir in 2000, according to the Centers for Disease Control and Prevention, the rates of primary and secondary syphilis as well as the total number of syphilis cases have been increasing steadily every year since then including an 11.2% increase from 2018 to 2019 [4,5]. Among the various groups at risk for syphilis are those who are positive for HIV. In fact, over 38% of early syphilis cases are reported with HIV coinfection [6,7]. Much like syphilis, however, HIV infection has many manifestations that can affect a wide range of different organ systems and predispose patients to infection or malignancy including lymphoma [8]. In this report, we present the case of an HIV-positive male who presented with unilateral tonsillar hypertrophy and ipsilateral lymphadenopathy with radiographic and pathologic findings concerning for lymphoma; however, upon surgical pathologic confirmation of an open biopsy of his right neck mass, he was diagnosed with secondary syphilis.

## Case presentation

**Figure 1:**
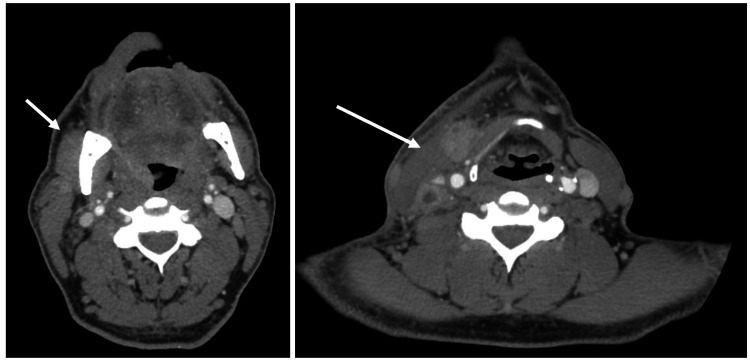
Cervical lymphadenopathy as shown by axial CT angiogram of the neck. Lymphadenopathy is shown by arrows in images.

A 48-year-old male with a long-standing history of HIV that was well-controlled with daily dolutegravir 50 mg and emtricitabine-tenofovir 200-25 mg was referred to head and neck surgery by his primary care provider for evaluation of oropharyngeal malignancy. The patient had been experiencing several symptoms for about a month before presentation to our clinic. He endorsed a 10-pound weight loss in the context of right tonsillar swelling and ipsilateral cervical lymphadenopathy accompanied by tonsillar pain that radiated to his right ear. The patient’s most recent CD4+ count was 686 cells/mm^3^ and viral load was <20 copies/mL of blood by polymerase chain reaction five months before presentation. A CT scan with contrast showed a right palatine tonsil measuring 1.3 × 0.8 cm with suspicion for neoplasm, accompanied by pathologic-appearing, right-sided level 2 and 3 lymph nodes measuring 2.0 × 0.8 cm and 2.0 × 1.5 cm, respectively, concerning for metastases (Figure 1). Socially, the patient reported a 70-pack-year tobacco smoking history with recently decreased use starting this year, as well as a history of weekly cocaine use discontinued in 2018. He did not currently drink alcohol but endorsed alcohol use until 2019. His medical history was also positive for chronic obstructive pulmonary disease and cardiovascular disease, as well as myocardial infarction with pacemaker placement in 2017. His most recent lab work revealed a non-reactive *T. pallidum* antibody titer five months before his initial consult visit.

**Figure 2:**
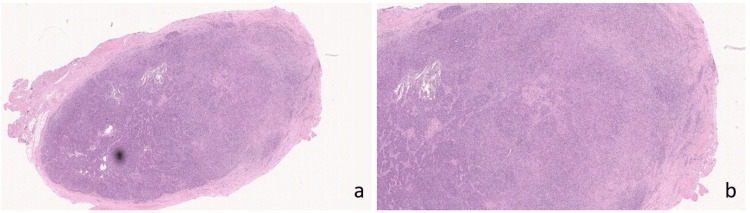
(a) Lymph node hematoxylin and eosin: lymph node shows partially effaced architecture and follicular hyperplasia at 10× magnification. (b) The lymph node shows an area of effacement containing syphilis organisms at 20× magnification.

**Figure 3:**
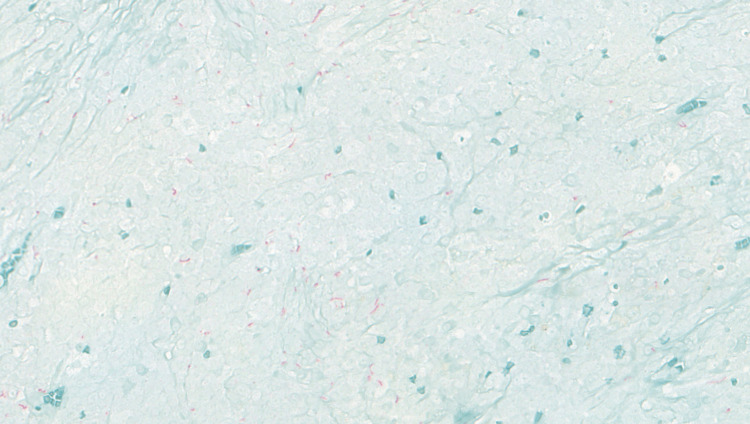
Treponema pallidum stain applied to lymph node tissue showing the presence of Treponema pallidum at 40× magnification.

On initial presentation, the patient was well-appearing with an examination significant for a 2+ right tonsil with medial ulceration and 3 × 3 cm right level 2 neck mass that was mildly tender to palpation. Flexible nasolaryngoscopy revealed no abnormalities other than the aforementioned tonsillar findings. The patient then underwent an in-office biopsy of the right tonsil, which he tolerated well. Surgical pathology of the biopsy tissue showed markedly inflamed tonsillar mucosa with both lymphoplasmacytic-predominant chronic and focally acute inflammation and prominent granulation tissue reaction. One week later, the patient returned to the clinic for fine-needle aspiration of the right neck mass that revealed atypical lymphoid proliferation consistent with lymphoma. The patient was then scheduled for an open biopsy in the operating room. A lymph right neck grossly enlarged lymph node was removed and sent as a fresh specimen to evaluate for lymphoma. The biopsy and surgical follow-up were uncomplicated. Hematologic pathology was positive for the spirochete *T. pallidum* with partially effaced lymph node architecture and follicular hyperplasia and no morphologic evidence of lymphoma (Figures 2, 3). Flow cytology of the mass showed 98% lymphocytes (26% B cells and 63% T cells) with no evidence of malignancy. T cells also showed an appropriate expression of lineage-specific markers with the CD4+/CD8+ of 40/25.

The pathologic results were communicated to the patient’s primary care provider as well as his case manager via telephone and facsimile. The patient was eventually reached with some difficulty and was informed of syphilis-positive biopsy results over the phone. The patient was given benzathine penicillin G 2.4 million units intramuscularly in a single dose by his primary care provider and was asked to follow up at the head and neck surgery office in two months. Unfortunately, the patient was lost to follow-up and did not come for a post-treatment examination.

## Discussion

In this report, we presented the case of a 48-year-old man with a long-standing history of well-controlled HIV who was referred to our head and neck clinic with unilateral tonsillar hypertrophy, cervical lymphadenopathy, facial pain, recent unexplained weight loss, and radiographic findings concerning for metastatic lymphoma. The patient underwent an in-office tonsillar biopsy and fine-needle aspiration of an enlarged neck lymph node that further raised suspicion for malignant lymphoma, particularly in the context of his chronic HIV infection, which led to an open biopsy in the operating room. Surgical pathology of the open biopsy sample revealed *T. pallidum* and the patient was consequentially diagnosed with secondary syphilis. This sexually transmitted infection has been steadily rising in prevalence in the United States in the last two decades. It disproportionately affects various unique patient populations, such as those diagnosed with HIV [5-7].

Among other groups, Komeno et al. presented a similar case of a younger man who presented with tonsillitis and cervical lymphadenopathy in addition to other markers of infection with secondary syphilis such as lung lesions and elevated liver enzymes [3]. However, this case from the Tokyo Yamate Medical Center is different from our case in multiple ways. First, the patient in their report presented with more features indicative of secondary syphilis than our patient, such as lung infection and elevated liver enzymes. Our patient presented with tonsillar hypertrophy and ulceration, lymphadenopathy, and recent weight loss which could possibly be attributed to either dysphagia due to his tonsillar manifestation or to secondary syphilis. However, his examination and laboratory values were otherwise normal. Additionally, our patient had a history of chronic HIV infection which predisposed him to several possible etiologies as the cause of his presentation, namely, a malignant and potentially metastatic lymphoma [8]. Lastly, the patient presented by the group in Japan had a different risk factor associated with an increased risk of contraction of syphilis, as he was a male who had sex with men (MSM). While both HIV-positive patients and MSM are disproportionately affected by syphilis infection, identification as an MSM in the absence of low CD4+ counts or low CD4+/CD8+ ratios does not independently increase the risk for pathologies such as lymphoma that commonly present in HIV-positive patients [1,9,10].

Chronic HIV is known to predispose patients to pathologies and malignancies ranging from opportunistic bacterial and fungal infections to various types of non-Hodgkin lymphoma (NHL) and, less commonly, Hodgkin lymphoma by the mechanism of decreasing cellular immunity. In fact, NHL, a group of lymphoproliferative malignancies that those with HIV are at a 60- to 200-fold increased risk of developing, is the leading cause of death in this patient population [11,12]. In patients with HIV, lymphoma commonly presents as an advanced disease with features such as weight loss, lymphadenopathy with extranodal involvement, and general malaise [12]. Thus, the recent subacute and unattributable 10-pound weight loss, physical examination findings, and recent imaging in this patient was concerning for malignancy.

While there are many manifestations of secondary syphilis involving various organ systems such as respiratory, gastrointestinal, renal, and dermatologic systems [1], not much has been published on the easily overlooked oral/oropharyngeal presentations of this infection. Lampros et al. described the two main oropharyngeal manifestations of secondary syphilis as those with ulcerative mucosal lesions (as seen in our patient’s tonsil) and mucous patches on the tongue, where ulcerative lesions were seen in 55% of patients with oral syphilis [9]. Lesions such as this that are concerning for secondary syphilis may often be accompanied by other less specific signs of infection, including the lymphadenopathy and weight loss seen in our patient [1]. As evidenced by the manifestations described above, many of the presenting symptoms seen in our patient pointed toward a diagnosis of malignant lymphoma. However, on further examination, it became clear that an infectious process was also highly possible due to patient history and biopsy findings. Syphilis was not initially a leading diagnosis because the patient followed up regularly with his primary care provider and case manager. He had an extensive history of negative syphilis screening tests, and CD4+ counts and viral loads within normal limits. His last documented labs within normal limits were less than five months before his presentation to our clinic. Thus, clinical suspicion and pathologic correlation were crucial in not only ruling out malignancy but also delineating the correct diagnosis.

A long-acting benzathine benzylpenicillin is the preferred and most commonly recommended drug for syphilis treatment [1,9]. For the majority of patients, one injection of 2.4 MU or two injections of 1.2 MU of penicillin in one session is the recommended dosing [1].^ ^With this dosing, particularly in patients with early disease and oral manifestations such as ours, outcomes are favorable within one to two weeks of therapy initiation [9]. The outcomes of patients diagnosed with syphilis who are treated with penicillin are so good in fact that even in those with penicillin allergy, the best option is to desensitize the patients so they can be treated with this medication. Although there is minimal and non-convincing data on the efficacy of alternative treatments for syphilis, doxycycline, ceftriaxone, and azithromycin may all be considered as choices instead of penicillin if the clinical situation demands [13]. In patients with an HIV coinfection, some studies have shown a potential benefit of increasing the dosage or duration of treatment with penicillin, whereas others have shown no benefit of increasing treatment dose or duration or even effective anti-retroviral therapy to be sufficient to decrease serologic rejection or disease progression [13].^ ^Treatment also varies based on the stage of infection, duration of infection, and presence or absence of neurologic symptoms [1]. These are all important considerations to make as future studies could be performed to better assess the efficacy of alternative treatments and the efficacy of varying penicillin regimens in patients with diagnosed HIV.

## Conclusions

Secondary syphilis can present in a myriad of different ways and can mimic several other pathologies depending on its manifestation. Therefore, particularly in instances where clinical suspicion is high for other etiologies, secondary syphilis could be missed. Oropharyngeal secondary syphilis should be considered as a possible diagnosis in the setting of tonsillar hypertrophy and lymphadenopathy concerning for lymphoma even in the setting of past negative syphilis tests, particularly when evaluating a patient with predisposing risk factors such as an HIV-positive status. It is imperative that this diagnosis is made, as if secondary syphilis can be identified, it is a readily treatable and potentially curable infection.
